# Clinical application of PIRCHE scores: reclassifying immunologic risk in patients with medium eplet mismatch

**DOI:** 10.3389/fimmu.2026.1873261

**Published:** 2026-07-30

**Authors:** Miklos Z. Molnar, Kendon J. Holdaway, Divya Raghavan, Silviana Marineci, Fruzsina Toth, Katalin Fornadi, Matthias Niemann

**Affiliations:** 1 Transplant Institute, University of Rochester Medical Center, Rochester, NY, United States; 2 Division of Nephrology, Department of Medicine, University of Rochester, Rochester, NY, United States; 3 Department of Surgery, University of Rochester, Rochester, NY, United States; 4 Department of Internal Medicine, Division of Nephrology & Hypertension, Spencer Fox Eccles School of Medicine at the University of Utah, Salt Lake City, UT, United States; 5 Doctoral College, Theoretical and Translational Medicine Division, Semmelweis University, Budapest, Hungary; 6 Department of Surgery, Division of Transplantation and Advanced Hepatobiliary Surgery, Spencer Fox Eccles School of Medicine at the University of Utah, Salt Lake City, UT, United States; 7 PIRCHE AG, Berlin, Germany

**Keywords:** antibody mediated rejection, donor derived cell free DNA, donor specific antibody, eplet mismatch, kidney transplantation, PIRCHE score, T cell mediated rejection

## Abstract

**Background:**

Molecular histocompatibility assessment using eplet mismatch and Predicted Indirectly ReCognizable HLA Epitopes (PIRCHE) scores has improved immunologic risk stratification in kidney transplantation, but its clinical implementation remains limited, particularly for donor–recipient pairs with medium eplet mismatch.

**Methods:**

We conducted a single-center retrospective cohort study of 493 adult kidney transplant recipients (2021–2024). Eplet mismatch was categorized based on Wiebe/Nickerson criteria, and medium mismatch pairs were further stratified using PIRCHE-T2 and PIRCHE-B scores. The primary outcome was early allograft injury within one year, defined as a composite of donor-specific antibody (DSA) development, histologic or molecular rejection, or elevation of donor-derived cell-free DNA (dd-cfDNA). Associations were assessed using Cox regression and Kaplan–Meier analyses.

**Results:**

Zero and low eplet mismatch groups had similar outcomes and were combined as the low-risk reference group. Compared with this group, medium eplet mismatch was associated with increased risk of early allograft injury (adjusted hazard ratio [aHR] 2.44, 95% confidence interval [CI] 1.41–4.23), biopsy-conditioned rejection (aHR 7.47, 95% CI 2.07–26.91), and elevated dd-cfDNA (aHR 4.83, 95% CI 1.70–13.75), while high mismatch conferred greater risk (aHR 5.17, 95% CI 2.86–9.33). Among medium mismatch recipients, ~20% were reclassified as medium–low risk based on PIRCHE scores; this subgroup showed no statistically significant increase in risk relative to the low-risk group for early allograft injury (aHR 1.87, 95% CI 0.92–3.81), DSA, rejection, or dd-cfDNA, although confidence intervals were wide and a clinically meaningful increase in risk cannot be excluded.

**Conclusions:**

Medium eplet mismatch recipients with low PIRCHE scores had no statistically significant increase in risk compared with the zero/low mismatch group, although confidence intervals were wide. Molecular matching may help refine risk stratification and expand donor options, but the PIRCHE-T2/-B thresholds—derived from an overlapping cohort—require external validation before clinical use.

## Introduction

Kidney transplantation is the treatment of choice for patients with end-stage kidney disease. Despite significant improvements in short-term outcomes, long-term graft survival has not improved substantially over the past decades. Chronic alloimmune injury remains a major cause of graft loss, alongside infection (1). Achieving an optimal balance between under- and over-immunosuppression remains a central challenge in transplantation. Over the past decades, the definition of immunologic risk has evolved considerably. Early approaches focused on panel reactive antibody levels and sensitization history, followed by the identification of donor-specific antibodies (DSA) and conventional human leukocyte antigen (HLA) antigen matching. More recently, the field has shifted toward molecular histocompatibility assessment, including eplet mismatch analysis and PIRCHE (Predicted Indirectly ReCognizable HLA Epitopes) scores.

Eplets represent small structural configurations of amino acids on the surface of HLA molecules that can be recognized by antibodies. Quantification of donor–recipient eplet mismatch provides a more refined assessment of immunologic compatibility than traditional antigen-level matching. Across more than twenty kidney transplant cohorts comprising hundreds to thousands of recipients, eplet mismatch has been consistently associated with alloimmune outcomes (2–21).

PIRCHE-T2 is a computational algorithm that predicts donor-derived HLA peptides that can be processed and presented by recipient HLA class II molecules and recognized by CD4^+^ T cells through the indirect pathway of allorecognition. The algorithm quantifies these predicted epitopes into scores reflecting the recipient’s immunologic exposure to donor HLA–derived peptides. The PIRCHE-B matching algorithm (formerly called Snow) further characterizes HLA protein surface structure by integrating predictions of amino acid surface area (Snowflake) and repeated local protrusion rank (Snowball) (22, 23). Across multiple cohorts, higher PIRCHE-T2 scores consistently predict increased risk of *de novo* donor-specific antibody (dnDSA) development, independent of conventional HLA mismatch and often independent of B-cell epitope load (14, 15, 24–29). We recently identified clinically applicable cutoff values for both PIRCHE-T2 and PIRCHE-B scores to distinguish low- from high-immunologic-risk transplantations (30). These cutoffs were derived using a cohort that overlaps substantially with the present study population; the current analysis therefore represents an internal application of these previously derived thresholds rather than an independent, external validation.

Despite strong evidence supporting the use of eplet mismatch to refine donor–recipient histocompatibility assessment, its implementation in routine clinical practice remains limited. One notable example is the National Kidney Registry (NKR), which incorporated DR/DQ-based eplet mismatch into its allocation algorithm in 2020 as part of the “Kidney for Life” initiative. The NKR uses four eplet mismatch categories—zero, low, medium, and high—based on the original Wiebe/Nickerson classification (2). Although initially derived from relatively small cohorts (31), subsequent studies confirmed that these categories correlate with post-transplant dnDSA risk and can effectively stratify immunologic risk (32). As a result, the NKR has increased the proportion of zero and low eplet mismatch transplants in recent years (33).

While prioritizing zero and low eplet mismatch transplantation is desirable, several practical challenges limit its widespread application. Identifying optimal matches may prolong waiting times, increasing dialysis exposure. Moreover, the majority of donor–recipient pairs fall within the medium eplet mismatch category, potentially excluding otherwise suitable donors if allocation focuses solely on zero or low mismatch. Integrating PIRCHE scores may help address this limitation by further stratifying medium eplet mismatch pairs into lower- and higher-risk categories.

In this retrospective single-center study, we evaluated whether PIRCHE scores can refine immunologic risk classification within medium eplet mismatch donor–recipient pairs. Specifically, we assessed the association between PIRCHE-modified eplet mismatch categories and early kidney allograft injury using complementary biomarkers, including donor-derived cell-free DNA (dd-cfDNA), donor-specific antibodies, histologic rejection, and molecular rejection signatures assessed by the Molecular Microscope Diagnostic System (MMDx) (34). We hypothesized that medium eplet mismatch transplants with low PIRCHE scores would demonstrate similar risk of early graft injury as zero/low eplet mismatch transplants, whereas medium mismatch transplants with high PIRCHE scores would be associated with increased risk during the first post-transplant year.

## Materials and methods

### Data source and study population

This single-center, retrospective, observational study included all adult kidney transplant recipients who underwent transplantation at the University of Utah between January 1, 2021, and December 31, 2024. All patients were followed for up to one year post-transplantation. Donor-related data were collected, including graft type (living donor, deceased donation after brain death (DBD), or donation after circulatory death (DCD)), demographics, comorbidities, terminal serum creatinine, cause of death, and serologic characteristics.

Recipient-level data included demographics, cause of end-stage renal disease, anthropometric measures, dialysis vintage, prior transplant history, multi-organ transplantation status, serologic data, and transplant-related variables such as induction immunosuppression, HLA-related data, laboratory values, and other relevant clinical parameters. Protocol biopsies are not performed at our center; all kidney allograft biopsies were performed for clinical indications. Histopathologic findings and molecular rejection assessments using the MMDx were collected for all biopsies.

Immunosuppressive therapy consisted of induction with either a lymphocyte-depleting agent or basiliximab, followed by standard maintenance immunosuppression. Maintenance therapy included prednisolone in combination with either belatacept, tacrolimus or cyclosporine A (microemulsion formulation, Neoral), together with mycophenolate mofetil, azathioprine, or sirolimus. Choice of induction agent (lymphocyte-depleting agent versus basiliximab) and maintenance regimen was based on immunologic risk assessment at the time of transplantation (including degree of HLA mismatch, sensitization history, and DSA status), donor type, and treating physician discretion, per institutional protocol; PIRCHE scores were not available to clinicians during the study period and therefore did not inform these decisions. Our institutional rejection-treatment protocol followed the standard-of-care practice (corticosteroid pulse therapy as first-line treatment for T-cell-mediated rejection, with anti-thymocyte globulin reserved for steroid-resistant or higher-grade rejection, and plasmapheresis/IVIG, with or without rituximab, for antibody-mediated rejection).

During the study period, DSA testing was performed at 3, 6, 9, and 12 months post-transplant per institutional protocol or at the discretion of the treating provider. Similarly, dd-cfDNA measurements were obtained at 1, 2, 3, 4, 6, 9, and 12 months post-transplant per protocol or as clinically indicated. Data were extracted from electronic medical records and local transplant databases and managed using the Research Electronic Data Capture (REDCap) system hosted by the University of Utah. The study was approved by the University of Utah Institutional Review Board, which granted a waiver of informed consent (#IRB_00162331).

### Eplet mismatch load

HLA typing was performed using molecular methods, including genomic DNA amplification followed by sequence-based typing and/or sequence-specific oligonucleotide probe hybridization. The proportion of low-resolution typing for either donor or recipient was 14% for HLA-A, 14% for HLA-B, 15% for HLA-C, 4% for HLA-DRB1, 27% for HLA-DQA1, and 1% for HLA-DQB1. Cases without available high-resolution typing for donor and recipient were excluded from analysis.

Eplet mismatch loads were calculated using the HLA Eplet Registry web service (https://www.epregistry.com.br/; software version February 2025; IMGT database version 3.62, updated November 6, 2025). The HLA Eplet Registry is an online database established under the auspices of the 16th International HLA and Immunogenetics Workshop in 2012. It documents all theoretically defined eplets and their antibody verification status (35), with the aim of reflecting the eplet repertoire incorporated in the widely used HLAMatchmaker software (36).

Class II eplet mismatch load was based on donor–recipient mismatches at the HLA-DRB, HLA-DQA1, and HLA-DQB1 loci, allowing derivation of both single molecular eplet mismatches and total eplet load. Similarly to NKR categories, we defined zero eplet mismatch if DR = 0 and DQ = 0; low eplet mismatch if DR<7 and DQ<9, excluding the zero/zero case; medium eplet mismatch if DR ≥ 7 and DQ ≤ 14 or DR: 0–6 and DQ: 9-14; high eplet mismatch if DR: 0–22 and DQ: 15-31.

### PIRCHE scores

Further molecular matching was assessed using predicted indirectly recognizable HLA-derived T cell epitope loads (PIRCHE-T2) and the number of surface-accessible HLA amino acid mismatches (PIRCHE-B). PIRCHE-T2 and PIRCHE-B scores were calculated using donor–recipient mismatches at the HLA-A, HLA-B, HLA-C, HLA-DRB1, and HLA-DQB1 loci to derive both locus-specific and aggregate scores. The scores were calculated using the PIRCHE web service (www.pirche.com, v4.5, Frost 1.1/Snow 1.1/IMGT 3.54, binding rank threshold 30%, surface area threshold 0.26, protrusion threshold 0.68). The overall five-locus PIRCHE-T2 and PIRCHE-B scores were used in this study. We used a cut off of 53 points for PIRCHE-T2 (low risk <53 and high risk ≥ 53) and 11 points for PIRCHE-B scores (low risk <11 and high risk ≥ 11) according to our previous results (30).

### Exposure variables

We first compared the zero and low eplet mismatch categories and found no significant differences in outcomes between these groups (see Results section). Therefore, these two categories were merged for subsequent analyses. We then assessed the association between three eplet mismatch categories (zero/low vs. medium vs. high) and study outcomes.

For the primary analysis, the medium eplet mismatch category (risk groups based on PIRCHE-T2 and PIRCHE-B scores) was further stratified using PIRCHE scores, resulting in four immunologic risk groups:

- Low risk: zero or low eplet mismatch, regardless of PIRCHE score.
- Medium-low risk: medium eplet mismatch with low-risk scores for both PIRCHE-T2 and PIRCHE-B.
- Medium-high risk: medium eplet mismatch with high-risk scores for either PIRCHE-T2 or PIRCHE-B.
- High risk: high eplet mismatch, regardless of PIRCHE score.

As sensitivity analysis (risk groups based on PIRCHE-T2 score), the medium eplet mismatch category was further stratified using only PIRCHE-T2 score, resulting in four immunologic risk groups:

- Low risk: zero or low eplet mismatch, regardless of PIRCHE-T2 score.
- Medium-low risk: medium eplet mismatch with low-risk scores for PIRCHE-T2.
- Medium-high risk: medium eplet mismatch with high-risk scores for PIRCHE-T2.
- High risk: high eplet mismatch, regardless of PIRCHE-T2 score.

### Outcome variables

Four distinct endpoints were evaluated. The first endpoint was the development of *de novo* or recurrent (n=27) donor-specific antibodies. Recurrent preexisting DSA was defined as DSA that was present at the time of transplantation (mean fluorescence intensity (MFI) >2,000), or historically detected but below the cutoff at the time of transplantation and subsequently detected within one year post-transplantation with an MFI >2,000. *De novo* DSA was defined as DSA detected within one year after transplantation with an MFI >2,000 that had not been detected either historically or at the time of transplantation.

The second endpoint was the detection of any type of rejection within the first year after transplantation, based on histopathological evaluation. Kidney biopsies were performed only when clinically indicated and were interpreted according to the Banff 2019 classification.

The third endpoint was the diagnosis of any form of molecular rejection within the first year after transplantation, assessed using the MMDx (34). For the composite primary outcome and the secondary rejection endpoint, histologic and molecular rejection were combined into a single rejection category (i.e., the rejection endpoint was considered met if either histologic or molecular rejection was diagnosed); molecular rejection was assessed only among patients who underwent a clinically indicated biopsy (i.e., the MMDx subset) and was not analyzed as an independent outcome.

The fourth endpoint was elevation of dd-cfDNA at any time during the first year after transplantation. dd-cfDNA elevation was defined as either (a) an absolute dd-cfDNA value ≥1.0%, or (b) an absolute dd-cfDNA value ≥0.5% accompanied by a ≥61% relative increase compared with the immediately preceding measurement (37).

The primary endpoint of the study was the occurrence of any of the above events within 12 months after transplantation. Time-to-event was defined as the interval from the date of transplantation to the date of the first occurrence of any endpoint event. Secondary endpoints consisted of each of the four outcomes evaluated independently. We have also analyzed the development of *de novo* DSA as a sensitivity analysis.

### Statistical analysis

Patient characteristics were summarized using mean ± standard deviation (SD) or median with interquartile range (IQR) for continuous variables, and counts with percentages for categorical variables. Comparisons between groups were performed using the Student’s *t*-test or the Mann–Whitney *U* test for continuous variables, and the chi-square test for categorical variables, as appropriate.

Associations between eplet mismatch categories and immunologic risk groups and clinical outcomes were evaluated using Cox proportional hazards regression models and Kaplan–Meier survival analyses with log-rank testing. The proportional hazards assumption was assessed using scaled Schoenfeld residuals. Variables included in multivariable-adjusted models were selected *a priori* based on biological plausibility, published literature, and clinical relevance, and were required to be available in the study database. Adjustments were made for recipient characteristics (age, sex, race/ethnicity, body mass index at transplantation, cause of end-stage renal disease, dialysis vintage, calculated panel reactive antibody (cPRA) at allocation, history of prior kidney transplantation, and preemptive transplant status), donor characteristics (age, sex, race/ethnicity, and donor type [living vs. deceased]), and transplantation-related factors (induction regimen, cold ischemia time (CIT), cytomegalovirus risk status, and kidney-alone versus multiorgan transplantation).

All statistical tests were two-sided, and a *p* value <0.05 was considered statistically significant. Given the number of covariates included in the adjusted models, we report the total number of events for each outcome model: 167 events for early graft injury, 106 for DSA, 69 for rejection, and 76 for elevated dd-cfDNA (out of 493 patients), corresponding to approximately 9.8, 6.2, 4.1, and 4.5 events per covariate, respectively, for the 17-covariate adjusted models; the lower ratios for rejection and dd-cfDNA fall below conventional thresholds for model stability and are noted as a limitation. Statistical analyses were performed using STATA version 19 (StataCorp, College Station, TX).

## Results

### Patient characteristics

**Figure 1:**
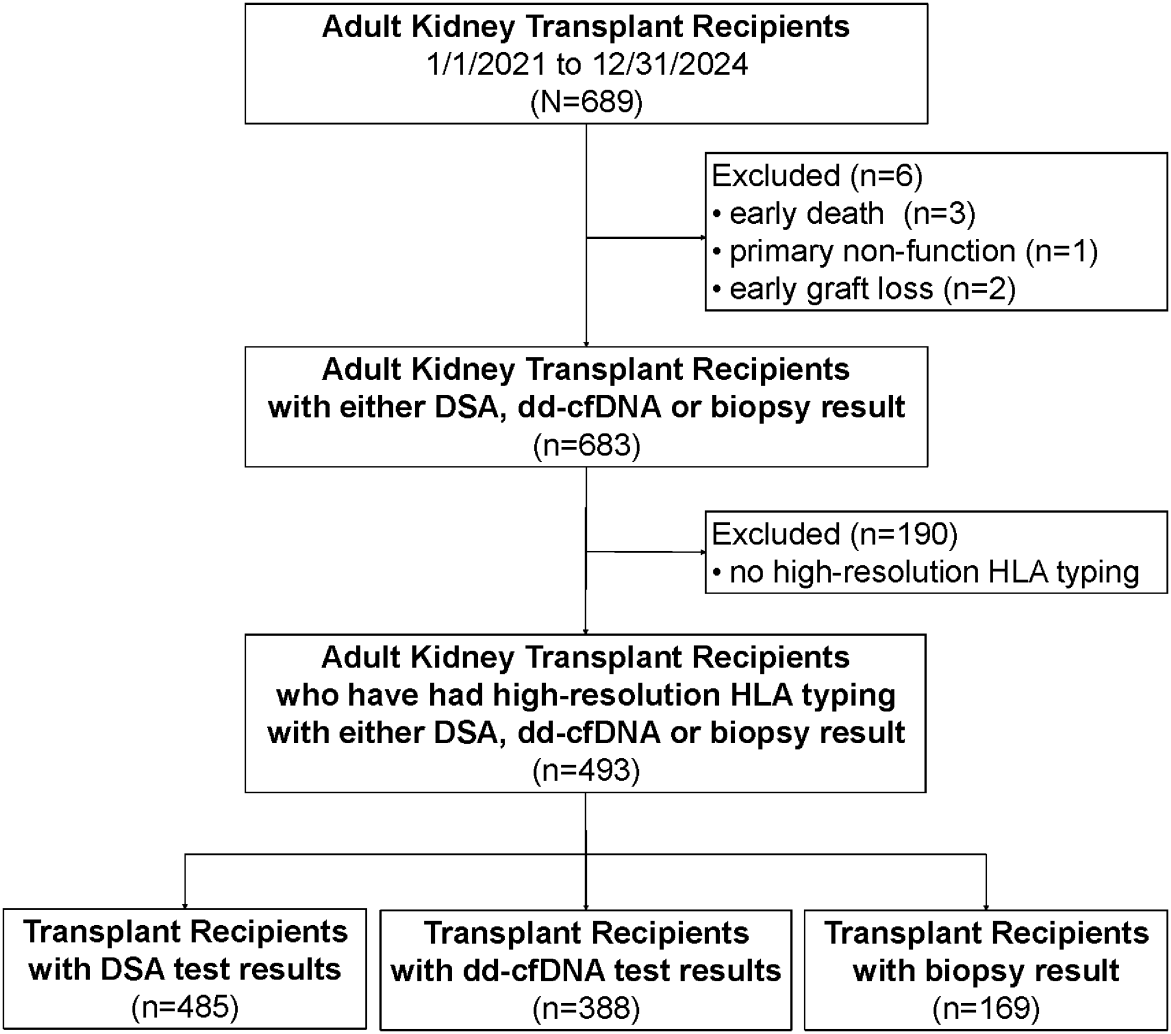
Flowchart of cohort selection.

Between January 1, 2021, and December 31, 2024, a total of 689 patients underwent kidney transplantation at the University of Utah and were initially included in the study cohort (**Figure 1**). Six patients were excluded due to missing outcome data resulting from early graft loss or death shortly after transplantation. Specifically, three patients died on postoperative days 6, 6, and 39; one experienced primary nonfunction of the allograft (donor quality); and two lost their grafts due to surgical complications (graft thrombosis) on postoperative days 1 and 2. Although these events were not considered alloimmune in nature, their exclusion may introduce survivorship bias by removing early technical and non-immunologic graft losses from the analytic cohort.

High-resolution HLA typing was unavailable in 190 patients; consequently, eplet mismatch and PIRCHE scores could not be calculated for these individuals. The final analytic cohort therefore consisted of 493 patients.

The mean (± SD) recipient age was 50 ± 15 years, and 61% were male. The cohort was racially and ethnically diverse, including 72% White, 18% Hispanic, 4% Asian, and 2% African American recipients. The median (IQR) dialysis vintage was 29 (14–50) months, and the mean (± SD) body mass index was 28.7 ± 5.6 kg/m². The median (IQR) cPRA was 0% (0%–0%), with a mean (± SD) of 14 ± 31%.

Living donor transplantation accounted for 41% of cases; among deceased donor transplants, 39% were donation after circulatory death. The mean (± SD) donor age was 37 ± 15 years; 54% of donors were male, and 78% were White. The median (IQR) kidney donor profile index (KDPI) was 23 (10–53), and the median (IQR) CIT was 12.1 (3.6–20.2) hours (**Table 1**).

**Table 1:** Patient characteristics based on eplet mismatch categories.

	Zero/low eplet mismatch	Medium eplet mismatch	High eplet mismatch	p-values
N	96	314	83	
Recipients’ characteristics				
Age (years), mean (SD)	49.7 (15.9)	49.9 (15.0)	49.1 (14.9)	0.92
Gender [N (%)]				<0.01
male	42 (43.8%)	205 (65.3%)	53 (63.9%)	
female	54 (56.2%)	109 (34.7%)	30 (36.1%)	
Race/ethnicity [N (%)]				0.25
White	80 (83.3%)	210 (70.0%)	54 (67.5%)	
Hispanic	12 (12.5%)	58 (19.3%)	15 (18.8%)	
African American	1 (1.0%)	9 (3.0%)	1 (1.2%)	
Asian	2 (2.1%)	12 (4.0%)	4 (5.0%)	
Other	1 (1.0%)	10 (3.3%)	6 (7.5%)	
Unknown	0 (0.0%)	1 (0.3%)	0 (0.0%)	
Body Mass Index (kg/m2), mean (SD)	28.0 (5.4)	28.9 (5.6)	28.8 (5.7)	0.35
Dialysis vintage (months), median (IQR)	24.5 (12.3-38.9)	30.0 (13.4-51.5)	34.4 (17.4-55.7)	0.20
Cause of kidney failure [N (%)]				0.04
Diabetes mellitus	17 (18%)	109 (35%)	21 (25%)	
Hypertension/Vascular disease	11 (11%)	38 (12%)	10 (12%)	
Cystic kidney disease	11 (11%)	25 (8%)	6 (7%)	
Glomerulonephritis	22 (23%)	69 (22%)	25 (30%)	
Other/unknown	35 (37%)	73 (23%)	21 (25%)	
Prior kidney transplant [N (%)]				<0.01
No	77 (80.2%)	289 (92.0%)	79 (95.2%)	
Yes	19 (19.8%)	25 (8.0%)	4 (4.8%)	
Multi-organ transplantation [N (%)]				0.07
Liver + kidney	0 (0.0%)	8 (2.5%)	4 (4.8%)	
Pancreas + kidney	0 (0.0%)	13 (4.1%)	2 (2.4%)	
Heart + kidney	1 (1.0%)	5 (1.6%)	4 (4.8%)	
None	95 (99.0%)	286 (91.1%)	72 (86.7%)	
Liver +Heart+Kidney	0 (0.0%)	2 (0.6%)	1 (1.2%)	
CMV risk categories [N (%)]				0.46
Low	19 (19.8%)	88 (28.0%)	15 (18.1%)	
Intermediate	47 (49.0%)	137 (43.6%)	41 (49.4%)	
High	27 (28.1%)	78 (24.8%)	25 (30.1%)	
Unknown	3 (3.1%)	11 (3.5%)	2 (2.4%)	
Was the donor organ pumped? [N (%)]				0.24
No	36 (40.9%)	112 (39.0%)	23 (29.5%)	
Yes	52 (59.1%)	175 (61.0%)	55 (70.5%)	
En block kidney [N (%)]	0 (0%)	6 (13%)	5 (33%)	0.04
Dual kidney [N (%)]	0 (0%)	5 (11%)	1 (7%)	0.47
Transplantation characteristics				
Cold ischemia time (hours), median (IQR)	12.6 (3.1-21.9)	11.0 (3.3-19.4)	14.8 (8.6-20.8)	0.04
cPRA (%), median (IQR)	0 (0-82)	0 (0-0)	0 (0-0)	<0.01
Donors’ characteristics				
Age (years), mean (SD)	37.6 (14.1)	37.9 (15.5)	34.7 (16.1)	0.24
Gender [N (%)]				0.08
male	43 (44.8%)	171 (54.5%)	51 (61.4%)	
female	53 (55.2%)	143 (45.5%)	32 (38.6%)	
Race/ethnicity				0.79
White	76 (79.2%)	245 (78.0%)	64 (77.1%)	
African American	2 (2.1%)	12 (3.8%)	4 (4.8%)	
Asian	2 (2.1%)	4 (1.3%)	3 (3.6%)	
Other	2 (2.1%)	6 (1.9%)	0 (0.0%)	
Unknown	14 (14.6%)	47 (15.0%)	12 (14.5%)	
Donor type [N (%)]				0.09
Living	42 (43.8%)	135 (43.0%)	25 (30.1%)	
Deceased	54 (56.2%)	179 (57.0%)	58 (69.9%)	
Donor DCD [N (%)]				0.59
No	31 (57.4%)	113 (63.1%)	33 (56.9%)	
Yes	23 (42.6%)	66 (36.9%)	25 (43.1%)	
KDPI, median (IQR)	28 (16-52)	22 (10-53)	26 (8-49)	0.46
Donor cause of death [N (%)]				0.42
Anoxia	27 (50.0%)	75 (41.9%)	29 (50.0%)	
Cerebrovascular/stroke	11 (20.4%)	22 (12.3%)	8 (13.8%)	
Head trauma	14 (25.9%)	73 (40.8%)	19 (32.8%)	
Central nervous system tumor	1 (1.9%)	1 (0.6%)	1 (1.7%)	
Other	1 (1.9%)	8 (4.5%)	1 (1.7%)	
Donor terminal creatinine (mg/dl), median (IQR)	0.78 (0.65-1.0)	0.82 (0.66-1.0)	0.83 (0.66-0.98)	0.72
Donors diabetes [N (%)]				0.63
No	91 (94.8%)	297 (96.1%)	81 (97.6%)	
Yes	5 (5.2%)	12 (3.9%)	2 (2.4%)	
Donors hypertension [N (%)]				0.92
No	85 (89.5%)	275 (89.3%)	72 (87.8%)	
Yes	10 (10.5%)	33 (10.7%)	10 (12.2%)	
Donor malignancy [N (%)]				0.29
No	95 (99.0%)	306 (97.5%)	79 (95.2%)	
Yes	1 (1.0%)	8 (2.5%)	4 (4.8%)	
Immunological characteristics				
Number of HLA mismatches (HLA A,B and DR) [N (%)]				<0.01
0	23 (24.0%)	0 (0.0%)	0 (0.0%)	
1	10 (10.4%)	4 (1.3%)	1 (1.2%)	
2	19 (19.8%)	14 (4.5%)	2 (2.4%)	
3	21 (21.9%)	50 (15.9%)	8 (9.6%)	
4	17 (17.7%)	81 (25.8%)	16 (19.3%)	
5	5 (5.2%)	106 (33.8%)	31 (37.3%)	
6	1 (1.0%)	59 (18.8%)	25 (30.1%)	
Induction treatment [N (%)]				
No Induction	0 (0.0%)	0 (0.0%)	0 (0.0%)	1.00
Basiliximab	17 (17.7%)	38 (12.1%)	11 (13.3%)	0.37
Thymoglobulin	73 (76.0%)	256 (81.5%)	70 (84.3%)	0.34
Steroid	96 (100.0%)	313 (99.7%)	83 (100.0%)	0.75
Alemtuzumab	4 (4.2%)	13 (4.1%)	1 (1.2%)	0.43
Outcomes				
Proportion of Antibody Mediated Rejection of the subset of patient underwent biopsy [N (%)]				0.01
No	24 (92.3%)	77 (71.3%)	20 (57.1%)	
Yes	2 (7.7%)	31 (28.7%)	15 (42.9%)	
Proportion of rejection of the subset of patient underwent biopsy [N (%)]				0.01
No	22 (84.6%)	61 (56.5%)	17 (48.6%)	
Yes	4 (15.4%)	47 (43.5%)	18 (51.4%)	
Proportion of T-cell Mediated Rejection of the subset of patient underwent biopsy [N (%)]				<0.01
No	24 (92.3%)	68 (63.0%)	20 (57.1%)	
Yes	2 (7.7%)	40 (37.0%)	15 (42.9%)	
Post-Transplant Donor Specific Antibodies [N (%)]				<0.01
No	76 (85.4%)	251 (80.2%)	52 (62.7%)	
Yes	13 (14.6%)	62 (19.8%)	31 (37.3%)	
Dd-cf-DNA elevation [N (%)]				<0.01
No	69 (94.5%)	198 (78.6%)	45 (71.4%)	
Yes	4 (5.5%)	54 (21.4%)	18 (28.6%)	
Primary outcome [N (%)]				<0.01
No	79 (82.3%)	210 (66.9%)	37 (44.6%)	
Yes	17 (17.7%)	104 (33.1%)	46 (55.4%)	
Delayed Graft Function [N (%)]				0.02
No	91 (94.8%)	291 (92.7%)	70 (84.3%)	
Yes	5 (5.2%)	23 (7.3%)	13 (15.7%)	
Death [N (%)]				0.28
No	92 (95.8%)	306 (97.5%)	78 (94.0%)	
Yes	4 (4.2%)	8 (2.5%)	5 (6.0%)	
Graft Loss [N (%)]				0.21
No	92 (95.8%)	304 (96.8%)	83 (100.0%)	
Yes	4 (4.2%)	10 (3.2%)	0 (0.0%)	

Values are expressed as mean (standard deviation), median (interquartile range), or number (%). Continuous variables were compared via t-tests or Mann-Whitney U tests. Categorical variables were compared via Chi-square tests.

Baseline recipient, donor, and transplant characteristics stratified by eplet mismatch category are presented in **Table 1**. Compared with patients in the zero/low eplet mismatch group, those in the higher eplet mismatch group were more likely to be male, more likely to receive en bloc kidneys, less likely to have undergone prior kidney transplantation, had longer cold ischemia times, and had a greater degree of HLA mismatching.

### Eplet mismatch groups and outcomes

Among the 493 patients included in the analysis, 48 had zero eplet mismatch, 48 had low mismatch, 314 had medium mismatch, and 83 had high mismatch. Kaplan–Meier survival analyses comparing the four eplet mismatch groups across all outcomes are shown in **Supplementary Figure 1**. The survival curves for the zero and low eplet mismatch groups were nearly overlapping, whereas the medium and high mismatch groups were clearly separated across all outcomes, suggesting similar risk profiles for the zero and low mismatch groups. These findings were supported by both unadjusted and adjusted Cox regression analyses (**Supplementary Table 1**). Compared with the zero mismatch group, the low mismatch group showed no significant differences in risk for any outcome (early graft injury, DSA, rejection, or elevated dd-cfDNA) (**Supplementary Table 1**). In contrast, both medium and high eplet mismatch groups were associated with increased risk in unadjusted and adjusted models (**Supplementary Table 1**). Given the similar risk profiles, the zero and low mismatch groups were combined for subsequent analyses.

After merging these categories, 96 patients had zero/low mismatch, 314 had medium mismatch, and 83 had high mismatch. Compared with the zero/low group, patients with medium eplet mismatch had a 2.4-fold higher risk of early graft injury (hazard ratio [HR] 2.44, 95% confidence interval [CI] 1.41–4.23), a 7.5-fold higher risk of rejection (HR 7.47, 95% CI 2.07–26.91), and a nearly 5-fold higher risk of elevated dd-cfDNA (HR 4.83, 95% CI 1.70–13.75) in unadjusted model (**Table 2**). There was also increased risk of post-transplant DSA (HR 1.73, 95% CI 0.89–3.32) in the unadjusted model, which was, however, not significant (**Table 2**).

**Table 2:** Association of three eplet mismatch categories and outcomes using Cox proportional regression models.

Unadjusted model												
Eplet MM	Early graft injury			DSA			Rejection			Elevated dd-cfDNA		
	HR	95%CI of HR	p	HR	95%CI of HR	p	HR	95%CI of HR	p	HR	95%CI of HR	p
Zero/Low	REFERENCE			REFERENCE			REFERENCE			REFERENCE		
Medium	1.94	1.16-3.24	0.01	1.29	0.71-2.34	0.41	3.17	1.14-8.82	0.03	4.12	1.49-11.37	<0.01
High	4.12	2.36-7.19	<0.01	2.96	1.55-5.66	<0.01	4.51	1.51-13.47	<0.01	5.74	1.94-16.95	<0.01
Adjusted model*												
Eplet MM	Early graft injury			DSA			Rejection			Elevated dd-cfDNA		
	HR	95%CI of HR	p	HR	95%CI of HR	p	HR	95%CI of HR	p	HR	95%CI of HR	p
Zero/Low	REFERENCE			REFERENCE			REFERENCE			REFERENCE		
Medium	2.44	1.41-4.23	<0.01	1.73	0.89-3.32	0.10	7.47	2.07-26.91	<0.01	4.83	1.70-13.75	<0.01
High	5.17	2.86-9.33	<0.01	3.72	1.85-7.47	<0.01	11.16	2.69-46.34	<0.01	5.81	1.92-17.64	<0.01

*adjusted for: recipient age, recipient gender, recipient race/ethnicity, recipient BMI, dialysis vintage, cause of end stage renal disease, preemptive kidney transplantation, history of prior kidney transplantation, multi-organ transplantation, type of induction therapy, donor age, donor gender, donor race/ethnicity, donor type [living vs. deceased], cold ischemia time, cytomegalovirus risk status, cPRA at allocation.

CI, confidence Interval; HR, Hazard Ratio.

MM, mismatch.

**Figure 2:**
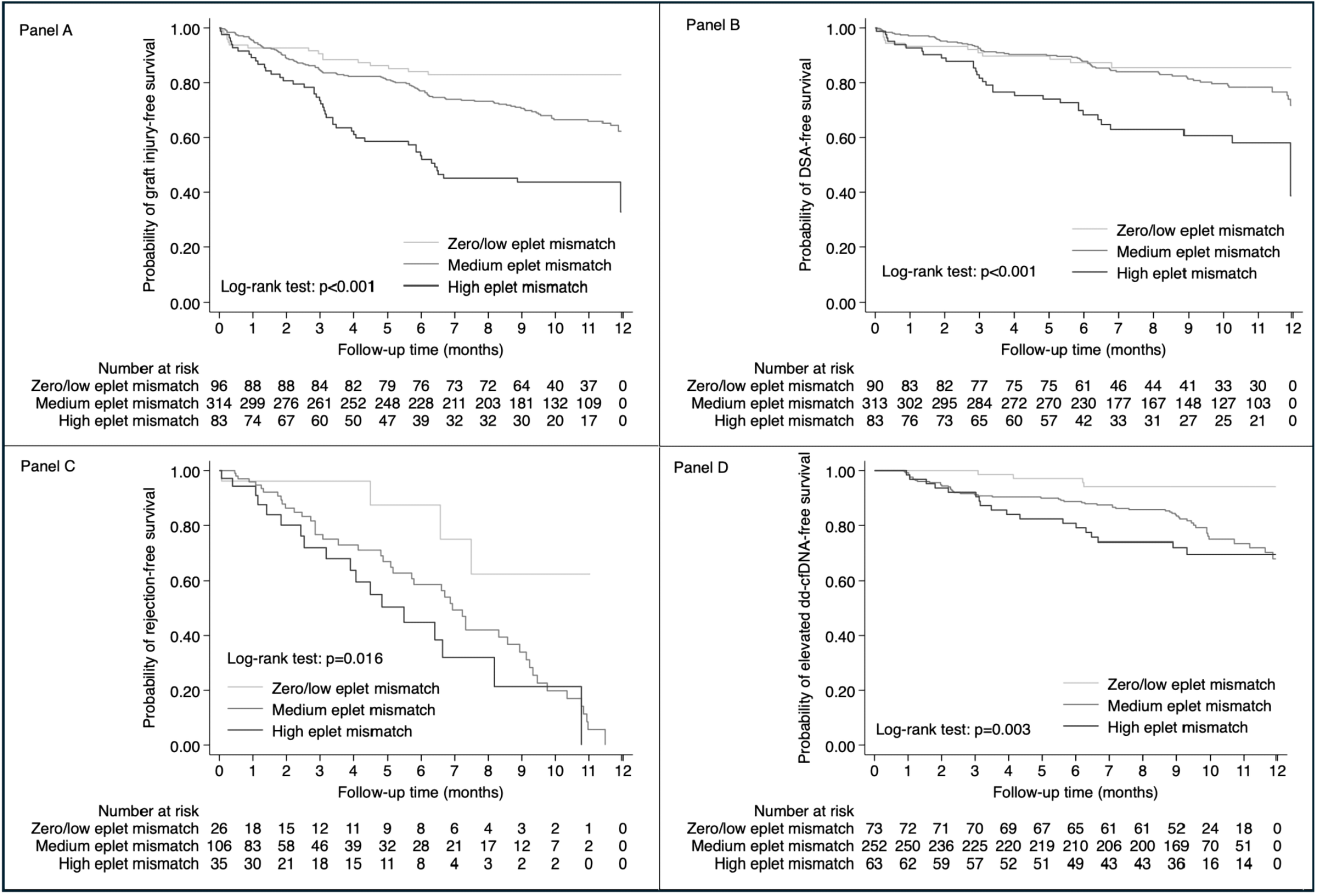
Kaplan-Meier curves by three Eplet groups for outcomes [Panel **(A)** Early Graft Injury outcome; Panel **(B)** DSA outcome; Panel **(C)** Rejection outcome; Panel **(D)** Elevation of dd-cfDNA outcome].

Patients with high eplet mismatch had an even greater risk compared with the zero/low group, including a 5.2-fold higher risk of early graft injury (HR 5.17, 95% CI 2.86–9.33), a 3.7-fold higher risk of post-transplant DSA (HR 3.72, 95% CI 1.85–7.47), an 11-fold higher risk of rejection (HR 11.16, 95% CI 2.69–46.34), and a nearly 6-fold higher risk of elevated dd-cfDNA (HR 5.81, 95% CI 1.92–17.64) in adjusted model (**Table 2**; **Figure 2**).

### Immunological risk groups and outcomes

#### Early allograft injury

**Figure 3:**
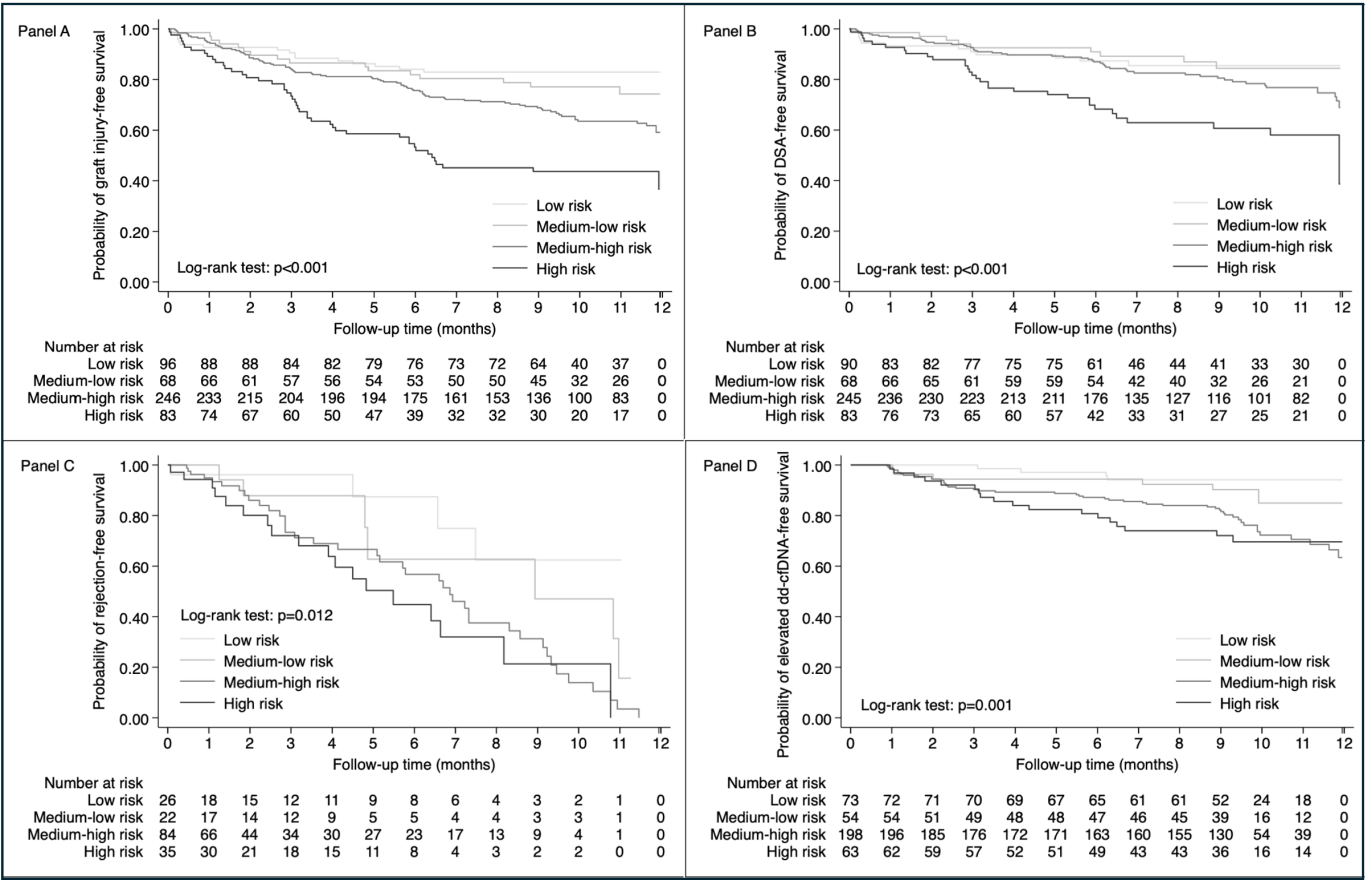
Kaplan-Meier curves by risk groups (risk groups based on PIRCHE-T2 and PIRCHE-B scores) for outcomes [Panel **(A)** Early Graft Injury outcome; Panel **(B)** DSA outcome; Panel **(C)** Rejection outcome; Panel **(D)** Elevation of dd-cfDNA outcome].

The 167 primary outcome events comprised 83 (50%) DSA events, 57 (34%) elevated dd-cfDNA events, and 27 (16%) rejection events. Among 96 patients with low immunological risk, 17 (18%) developed early allograft injury, compared with 16/68 (24%) in the medium-low, 88/246 (36%) in the medium-high, and 46/83 (55%) in the high-risk groups. In unadjusted analyses, patients with medium-low risk had a similar risk of early allograft injury compared with the low-risk group (HR 1.32, 95% CI 0.67–2.62), whereas medium-high (HR 2.12, 95% CI 1.26–3.56) and high-risk groups (HR 4.12, 95% CI 2.36–7.20) had significantly increased risk (**Table 3**). These findings remained consistent after adjustment for recipient, donor, and transplant-related factors (medium-low: HR 1.87, 95% CI 0.92–3.81; medium-high: HR 2.62, 95% CI 1.49–4.58; high: HR 5.20, 95% CI 2.89–9.38). Kaplan–Meier analyses (**Figure 3A**) demonstrated overlapping survival curves for the low and medium-low risk groups, whereas the medium-high and high-risk groups showed clear separation.

**Table 3:** Association of risk groups (risk groups based on PIRCHE-T2 and PIRCHE-B scores) and outcomes using Cox proportional regression models.

Unadjusted model												
Risk groups	Early graft injury			DSA			Rejection			Elevated dd-cfDNA		
	HR	95%CI of HR	p	HR	95%CI of HR	p	HR	95%CI of HR	p	HR	95%CI of HR	p
Low	REFERENCE			REFERENCE			REFERENCE			REFERENCE		
Medium-low	1.32	0.67-2.62	0.42	0.86	0.37-2.00	0.72	1.88	0.55-6.42	0.32	2.06	0.58-7.30	0.26
Medium-high	2.12	1.26-3.56	<0.01	1.41	0.77-2.58	0.27	3.67	1.30-10.35	0.01	4.71	1.70-13.05	<0.01
High	4.12	2.36-7.20	<0.01	2.96	1.55-5.66	<0.01	4.63	1.55-13.87	<0.01	5.74	1.94-16.95	<0.01
Adjusted model*												
Risk Groups	Early graft injury			DSA			Rejection			Elevated dd-cfDNA		
	HR	95%CI of HR	p	HR	95%CI of HR	p	HR	95%CI of HR	p	HR	95%CI of HR	p
Low	REFERENCE			REFERENCE			REFERENCE			REFERENCE		
Medium-low	1.87	0.92-3.81	0.09	1.22	0.47-2.98	0.67	3.36	0.73-15.50	0.12	2.57	0.72-9.23	0.15
Medium-high	2.62	1.49-4.58	<0.01	1.87	0.96-3.63	0.07	9.23	2.52-33.89	<0.01	5.91	2.04-17.13	<0.01
High	5.20	2.89-9.38	<0.01	3.74	1.86-7.50	<0.01	12.46	3.00-51.70	<0.01	6.10	2.01-18.56	<0.01

*adjusted for: recipient age, recipient gender, recipient race/ethnicity, recipient BMI, dialysis vintage, cause of end stage renal disease, preemptive kidney transplantation, history of prior kidney transplantation, multi-organ transplantation, type of induction therapy, donor age, donor gender, donor race/ethnicity, donor type [living vs. deceased], cold ischemia time, cytomegalovirus risk status, cPRA at allocation.

CI, confidence Interval; HR, Hazard Ratio.

#### Post-transplant donor-specific antibodies

Among patients with available data, 13/89 (15%) in the low-risk group developed post-transplant DSA, compared with 9/68 (23%) in the medium-low, 53/245 (22%) in the medium-high, and 31/83 (37%) in the high-risk groups. Kaplan–Meier analyses (**Figure 3B**) showed overlapping curves for the low and medium-low groups, with clear separation for the medium-high and high-risk groups. These findings were supported by both unadjusted and adjusted Cox regression analyses (**Table 3**).

#### Rejection

Cox regression models for the rejection endpoint were fit in the cohort that underwent kidney biopsy. Using the full-cohort denominators, rejection occurred in 4/96 (4.2%) of the low-risk group, 7/68 (10.3%) of the medium-low group, 40/246 (16.3%) of the medium-high group, and 18/83 (21.7%) of the high-risk group. Because kidney biopsies were performed only for clinical indications, and biopsy decisions may themselves have been influenced by dd-cfDNA, DSA, graft function, or clinician concern, the following rate should be interpreted as biopsy-conditioned (i.e., clinically detected biopsy-proven) rejection rather than as rejection risk across the entire cohort. Restricted to the subset of patients who underwent clinically indicated biopsy, biopsy-conditioned rejection was diagnosed in 4/26 (15%), 7/22 (32%), 40/86 (47%), and 18/35 (51%) of the low, medium-low, medium-high, and high-risk groups, respectively. Kaplan–Meier analyses (**Figure 3C**) and Cox regression models (**Table 3**) demonstrated similar risk between the low and medium-low groups, with significantly higher risk in the medium-high and high-risk groups.

#### Elevated donor-derived cell-free DNA

Among patients with available dd-cfDNA data, elevated levels were observed in 4/73 (5%) of the low-risk group, 6/54 (11%) of the medium-low group, 48/198 (24%) of the medium-high group, and 18/63 (29%) of the high-risk group. Kaplan–Meier analyses (**Figure 3D**) showed overlapping curves for the low and medium-low groups, with clear separation for the medium-high and high-risk groups. These findings were consistent with both unadjusted and adjusted Cox regression analyses (**Table 3**).

### Sensitivity analysis

The sensitivity analysis using PIRCHE-T2 alone to stratify risk within the medium eplet mismatch group yielded qualitatively similar results across all outcomes, as demonstrated by both unadjusted and adjusted Cox regression models (**Supplementary Table 2**) and Kaplan–Meier survival analyses (**Supplementary Figure 2**). Similar associations were found for the primary outcome and all secondary outcomes in our sensitivity analysis after excluding multiorgan recipients (not shown). The sensitivity analysis focusing on the development of *de novo* DSA showed qualitatively similar results (**Supplementary Tables 3–6**; **Supplementary Figure 3**).

## Discussion

In this single-center retrospective study of nearly 500 kidney transplant recipients, we demonstrate that patients with medium eplet mismatch—defined by Wiebe/Nickerson (2, 31, 32) and currently used in the NKR—and low PIRCHE-T2 and PIRCHE-B scores have a similar immunologic risk of early allograft injury during the first post-transplant year as those with zero or low eplet mismatch. By integrating multiple complementary and clinically validated biomarkers—including donor-specific antibodies, donor-derived cell-free DNA, histologic rejection, and molecular rejection assessed by the Molecular Microscope^®^ Diagnostic System—we provide a comprehensive assessment of early alloimmune injury. Notably, more than 20% of patients in the medium eplet mismatch group were reclassified into a lower-risk category, potentially expanding the pool of acceptable donors for patients requiring low-immunologic-risk transplantation.

There is an increasing need to refine and periodically update the definition of low immunologic risk in transplantation. Although the United Network for Organ Sharing continues to rely primarily on antigen-level HLA matching, molecular histocompatibility assessment is increasingly being incorporated into clinical practice. A prominent example is the NKR, which has integrated DR/DQ eplet mismatch into its allocation algorithm.

Transplant physicians frequently face situations in which, either due to patient preference or clinical considerations, it is necessary to select donor organs associated with the lowest immunologic risk. While this approach may be difficult to implement in deceased donor transplantation, it is commonly applied in living donor candidates participating in paired exchange programs. In this setting, identifying donors associated with low immunologic risk is particularly important for sensitized patients or those requiring tailored immunosuppression. However, restricting donor selection to zero or low eplet mismatch may significantly limit donor availability and prolong waiting times, especially in paired exchange programs. Our findings suggest that molecular refinement of immunologic risk using PIRCHE scores may help address this limitation.

First, we demonstrate that zero- and low-eplet mismatch transplantation is associated with similarly favorable outcomes, including early graft injury, donor-specific antibody development, and elevation of donor-derived cell-free DNA. These findings support that zero- and low-eplet mismatch categories have similar observed outcomes, as currently defined in NKR practice, and suggest that patients seeking “zero mismatch” transplantation may be considered in appropriately selected patients, thereby expanding their donor pool and potentially reducing waiting time. To our knowledge, this is the first study to directly evaluate and demonstrate comparable outcomes between zero- and low-eplet mismatch transplantation.

Second, we show that the medium eplet mismatch category is heterogeneous. Patients with medium eplet mismatch and low PIRCHE-T2 and PIRCHE-B scores showed no statistically significant increase in risk relative to those with zero/low eplet mismatch across all outcomes examined, although confidence intervals were wide (e.g., adjusted HR 1.87, 95% CI 0.92–3.81 for early graft injury; adjusted HR 3.36, 95% CI 0.73–15.50 for rejection) and a clinically meaningful increase in risk cannot be excluded; these findings should therefore be considered hypothesis-generating pending external validation. This is particularly important because the PIRCHE-T2 and PIRCHE-B cutoffs applied here were derived from a cohort that overlaps substantially with the present study population (30); this analysis therefore constitutes an internal application of previously derived thresholds rather than an independent validation. Importantly, medium eplet mismatch accounted for more than 60% of our cohort, and approximately 20% of these patients could be reclassified into a lower-risk group using PIRCHE scores. This finding has potential clinical implications as a candidate strategy to expand the pool of low immunological risk donor options in both living and deceased donor transplantation, pending prospective validation in independent, more highly sensitized, multi-center cohorts with longer follow-up. Thus, this study provides a candidate framework for implementing PIRCHE scores in clinical practice. Lastly, the data also indicates an increased proportion of immunological low risk transplant recipients, that may benefit from individualized immunosuppressive strategies.

A key strength of this study is the use of a composite outcome integrating serologic, molecular, and histologic markers of injury. This approach reflects real-world clinical practice, where allograft injury is multifactorial and may not be fully captured by a single biomarker. Our findings suggest that molecular histocompatibility assessment, particularly when combining eplet mismatch and PIRCHE scores, captures a global propensity for alloimmune injury rather than a single downstream manifestation. At the same time, the number of events relative to the number of covariates was limited for the rejection and dd-cfDNA models in particular (approximately 4.1 and 4.5 events per covariate, respectively); these secondary-outcome analyses should therefore be interpreted as exploratory and potentially unstable, and replication in larger, external cohorts is needed.

Several limitations should be considered. The retrospective, single-center design limits causal inference and generalizability, and external validation in independent and more diverse cohorts is warranted. In addition, the PIRCHE-T2 and PIRCHE-B cutoffs applied in this study were derived from a cohort that overlaps substantially with the present population (30); therefore, this study should be regarded as an internal, exploratory application of these thresholds rather than an independent validation, and prospective external validation in separate cohorts is required before clinical adoption. The population was also relatively unsensitized overall (median cPRA 0%), and follow-up was limited to one year; these findings should therefore be considered a candidate risk-stratification strategy rather than a ready-to-implement allocation rule, pending prospective, longer-term, multi-center validation. Prospective validation in independent, more highly sensitized, multi-center cohorts with longer follow-up is also required before this approach could inform donor allocation or organ-offer decisions. The medium-low risk subgroup was relatively small (n=68), and the corresponding confidence intervals were wide (e.g., adjusted HR 1.87, 95% CI 0.92–3.81 for early graft injury; adjusted HR 3.36, 95% CI 0.73–15.50 for rejection); absence of statistical significance in this subgroup does not establish equivalence with the zero/low mismatch group, and a clinically meaningful increase in risk cannot be excluded. We also note that the rejection endpoint relied on clinically indicated rather than protocol biopsies, so this outcome is subject to potential ascertainment bias if biopsy decisions were influenced by dd-cfDNA, DSA, graft function, or clinician concern, and that the number of events relative to the number of covariates in the adjusted models was limited for the rejection and dd-cfDNA endpoints in particular, raising concern for model instability. We did not systematically account for differences in immunosuppressive regimens, nor did we systematically capture post-transplant infectious complications (e.g., BK viremia, CMV reactivation) or immunosuppressive drug levels, which may independently influence alloimmune outcomes. Another potential limitation of this study is selection bias due to differences between included and excluded recipients (**Supplementary Table 7**). Excluded patients had characteristics associated with higher transplant risk, including longer dialysis vintage, greater use of deceased donor kidneys, higher KDPI donors, longer cold ischemia times, and higher mortality, whereas the included cohort contained a greater proportion of lower-risk living donor recipients. Therefore, the findings may have limited generalizability to higher-risk kidney transplant populations. However, PIRCHE scores were not used to guide clinical decision-making in this cohort, making it unlikely that treatment bias significantly influenced our findings. As with all observational studies, residual confounding cannot be excluded despite multivariable adjustment.

Despite these limitations, the strengths of this study—including comprehensive biomarker assessment, robust statistical modeling, and a clinically applicable framework—support the translational relevance of our findings. Molecular histocompatibility assessment provides a practical and scalable approach to refine immunologic risk stratification and can be readily integrated into existing transplant workflows, particularly in paired exchange programs.

In conclusion, transplantation with zero or low eplet mismatch, as well as medium eplet mismatch with low PIRCHE scores, was associated with no statistically significant increase in immunologic risk during the first year after kidney transplantation, although confidence intervals for the medium-low risk group were wide. These findings provide a candidate framework for reclassifying immunologic risk and, pending external and prospective validation, may inform future clinical trials aimed at individualized immunosuppression and optimization of donor selections.

## Data Availability

The dataset is owned by the University. Requests to access the datasets should be directed to MM: Miklos_Molnar@urmc.rochester.edu.
