# Supplementary material for: Clinical application of PIRCHE scores: reclassifying immunologic risk in patients with medium eplet mismatch
Source: Front Immunol. 2026 Jul 30;17:1873261. doi: 10.3389/fimmu.2026.1873261 (PMC13469082; doi:10.3389/fimmu.2026.1873261)
Supplement: Supplementary file 1 [file SupplementaryFile1.docx]

**Clinical Application of PIRCHE Scores: Reclassifying Immunologic Risk in Patients with Medium Eplet Mismatch**

**Supplementary Materials**

**Supplementary Tables**

**Supplemental Table 1:** Association of four Eplet mismatch categories and outcomes using Cox proportional regression models

**Supplemental Table 2:** Association of risk groups (risk groups based on PIRCHE-T2) and outcomes using Cox proportional regression models

**Supplemental Table 3:** Association of three Eplet mismatch categories and developing de novo DSA using Cox proportional regression models

**Supplemental Table 4:** Association of risk groups (risk groups based on PIRCHE-T2 and PIRCHE-B scores) and developing de novo DSA using Cox proportional regression models

**Supplemental Table 5:** Association of four Eplet mismatch categories and developing de novo DSA using Cox proportional regression models

**Supplemental Table 6:** Association of risk groups (risk groups based on PIRCHE-T2) and developing de novo DSA using Cox proportional regression models

**Supplemental Table 7:** Baseline characteristics of excluded versus included patients in the whole cohort

**Supplemental Table 1:** Association of four Eplet mismatch categories and outcomes using Cox proportional regression models

| **Unadjusted model** | | | | | | | | | | | | |
| --- | --- | --- | --- | --- | --- | --- | --- | --- | --- | --- | --- | --- |
| **Eplet MM** | **Early Graft Injury** | | | **DSA** | | | **Rejection** | | | **Elevated dd-cfDNA** | | |
|  | **HR** | **95%CI of HR** | **p** | **HR** | **95%CI of HR** | **p** | **HR** | **95%CI of HR** | **p** | **HR** | **95%CI of HR** | **p** |
| Zero | REFERENCE | | | REFERENCE | | | REFERENCE | | | REFERENCE | | |
| Low | 0.84 | 0.32-2.18 | 0.72 | 0.67 | 0.23-2.01 | 0.48 | 3.81 | 0.40-36.66 | 0.25 | 2.26 | 0.24-21.76 | 0.48 |
| Medium | 1.78 | 0.90-3.52 | 0.10 | 1.05 | 0.48-2.30 | 0.90 | 7.08 | 0.98-51.39 | 0.05 | 7.09 | 0.98-51.23 | 0.05 |
| High | 3.78 | 1.85-7.73 | <0.01 | 2.42 | 1.07-5.50 | 0.04 | 10.09 | 1.34-75.92 | 0.03 | 9.87 | 1.32-73.92 | 0.03 |
| **Adjusted model*** | | | | | | | | | | | | |
| **Eplet MM** | **Early Graft Injury** | | | **DSA** | | | **Rejection** | | | **Elevated dd-cfDNA** | | |
|  | **HR** | **95%CI of HR** | **p** | **HR** | **95%CI of HR** | **p** | **HR** | **95%CI of HR** | **p** | **HR** | **95%CI of HR** | **p** |
| Zero | REFERENCE | | | REFERENCE | | | REFERENCE | | | REFERENCE | | |
| Low | 1.11 | 0.42-2.96 | 0.83 | 1.08 | 0.35-3.31 | 0.90 | 13.61 | 1.14-162.3 | 0.04 | 2.16 | 0.22-21.01 | 0.51 |
| Medium | 2.57 | 1.23-5.35 | 0.01 | 1.78 | 0.76-4.23 | 0.19 | 33.83 | 3.62-316.5 | <0.01 | 8.11 | 1.10-59.89 | 0.04 |
| High | 5.44 | 2.53-11.71 | <0.01 | 3.85 | 1.57-9.45 | <0.01 | 52.35 | 5.01-546.6 | <0.01 | 9.77 | 1.27-74.94 | 0.03 |

*adjusted for: recipient age, recipient gender, recipient race/ethnicity, recipient BMI, dialysis vintage, cause of end stage renal disease, preemptive kidney transplantation, history of prior kidney transplantation, multi-organ transplantation, type of induction therapy, donor age, donor gender, donor race/ethnicity, donor type [living vs. deceased], cold ischemia time, cytomegalovirus risk status, cPRA at allocation

Abbreviations:

CI: confidence Interval

HR: Hazard Ratio

MM: mismatch

**Supplemental Table 2:** Association of risk groups (risk groups based on PIRCHE-T2) and outcomes using Cox proportional regression models

| **Unadjusted model** | | | | | | | | | | | | |
| --- | --- | --- | --- | --- | --- | --- | --- | --- | --- | --- | --- | --- |
| **Risk Groups** | **Early Graft Injury** | | | **DSA** | | | **Rejection** | | | **Elevated dd-cfDNA** | | |
|  | **HR** | **95%CI of HR** | **p** | **HR** | **95%CI of HR** | **p** | **HR** | **95%CI of HR** | **p** | **HR** | **95%CI of HR** | **p** |
| Low | REFERENCE | | | REFERENCE | | | REFERENCE | | | REFERENCE | | |
| Medium-low | 1.50 | 0.82-2.71 | 0.19 | 0.90 | 0.43-1.88 | 0.78 | 2.18 | 0.71-6.70 | 0.17 | 3.03 | 1.01-9.13 | 0.05 |
| Medium-high | 2.20 | 1.30-3.73 | <0.01 | 1.51 | 0.82-2.80 | 0.19 | 3.97 | 1.39-11.35 | 0.01 | 4.78 | 1.71-13.38 | <0.01 |
| High | 4.12 | 2.36-7.19 | <0.01 | 2.96 | 1.55-5.66 | <0.01 | 4.69 | 1.56-14.09 | <0.01 | 5.74 | 1.94-16.96 | <0.01 |
| **Adjusted model*** | | | | | | | | | | | | |
| **Risk Groups** | **Early Graft Injury** | | | **DSA** | | | **Rejection** | | | **Elevated dd-cfDNA** | | |
|  | **HR** | **95%CI of HR** | **p** | **HR** | **95%CI of HR** | **p** | **HR** | **95%CI of HR** | **p** | **HR** | **95%CI of HR** | **p** |
| Low | REFERENCE | | | REFERENCE | | | REFERENCE | | | REFERENCE | | |
| Medium-low | 2.04 | 1.09-3.82 | 0.03 | 1.26 | 0.58-2.76 | 0.56 | 4.46 | 1.10-18.09 | 0.04 | 3.61 | 1.17-11.17 | 0.03 |
| Medium-high | 2.68 | 1.52-4.74 | <0.01 | 1.98 | 1.01-3.88 | 0.05 | 10.92 | 2.85-41.85 | <0.01 | 5.89 | 2.01-17.26 | <0.01 |
| High | 5.20 | 2.88-9.38 | <0.01 | 3.73 | 1.86-7.48 | <0.01 | 13.62 | 3.25-57.10 | <0.01 | 6.03 | 1.98-18.36 | <0.01 |

*adjusted for: recipient age, recipient gender, recipient race/ethnicity, recipient BMI, dialysis vintage, cause of end stage renal disease, preemptive kidney transplantation, history of prior kidney transplantation, multi-organ transplantation, type of induction therapy, donor age, donor gender, donor race/ethnicity, donor type [living vs. deceased], cold ischemia time, cytomegalovirus risk status, cPRA at allocation

Abbreviations:

CI: confidence Interval

HR: Hazard Ratio

**Supplemental Table 3:** Association of three Eplet mismatch categories and developing de novo DSA using Cox proportional regression models

| **Unadjusted model** | | | |
| --- | --- | --- | --- |
| **Eplet MM** | **De novo DSA** | | |
|  | **HR** | **95%CI of HR** | **p** |
| Zero/Low | REFERENCE | | |
| Medium | 1.64 | 0.81-3.32 | 0.17 |
| High | 3.65 | 1.71-7.79 | <0.01 |
| **Adjusted model*** | | | |
| **Eplet MM** | **De novo DSA** | | |
|  | **HR** | **95%CI of HR** | **p** |
| Zero/Low | REFERENCE | | |
| Medium | 2.07 | 0.97-4.44 | 0.06 |
| High | 4.24 | 1.88-9.53 | <0.01 |

*adjusted for: recipient age, recipient gender, recipient race/ethnicity, recipient BMI, dialysis vintage, cause of end stage renal disease, preemptive kidney transplantation, history of prior kidney transplantation, multi-organ transplantation, type of induction therapy, donor age, donor gender, donor race/ethnicity, donor type [living vs. deceased], cold ischemia time, cytomegalovirus risk status, cPRA at allocation

Abbreviations:

CI: confidence Interval

HR: Hazard Ratio

MM: mismatch

**Supplemental Table 4:** Association of risk groups (risk groups based on PIRCHE-T2 and PIRCHE-B scores) and developing de novo DSA using Cox proportional regression models

| **Unadjusted model** | | | |
| --- | --- | --- | --- |
| **Risk Groups** | **De novo DSA** | | |
|  | **HR** | **95%CI of HR** | **p** |
| Low | REFERENCE | | |
| Medium-low | 1.09 | 0.42-2.82 | 0.86 |
| Medium-high | 1.80 | 0.88-3.66 | 0.11 |
| High | 3.65 | 1.71-7.79 | <0.01 |
| **Adjusted model*** | | | |
| **Risk Groups** | **De novo DSA** | | |
|  | **HR** | **95%CI of HR** | **p** |
| Low | REFERENCE | | |
| Medium-low | 1.45 | 0.53-3.95 | 0.47 |
| Medium-high | 2.24 | 1.04-4.85 | 0.04 |
| High | 4.25 | 1.89-9.55 | <0.01 |

*adjusted for: recipient age, recipient gender, recipient race/ethnicity, recipient BMI, dialysis vintage, cause of end stage renal disease, preemptive kidney transplantation, history of prior kidney transplantation, multi-organ transplantation, type of induction therapy, donor age, donor gender, donor race/ethnicity, donor type [living vs. deceased], cold ischemia time, cytomegalovirus risk status, cPRA at allocation

Abbreviations:

CI: confidence Interval

HR: Hazard Ratio

**Supplemental Table 5:** Association of four Eplet mismatch categories and developing de novo DSA using Cox proportional regression models

| **Unadjusted model** | | | |
| --- | --- | --- | --- |
| **Eplet MM** | **De novo DSA** | | |
|  | **HR** | **95%CI of HR** | **p** |
| Zero | REFERENCE | | |
| Low | 0.97 | 0.26-3.63 | 0.97 |
| Medium | 1.62 | 0.59-4.46 | 0.35 |
| High | 3.59 | 1.25-10.30 | 0.02 |
| **Adjusted model*** | | | |
| **Eplet MM** | **De novo DSA** | | |
|  | **HR** | **95%CI of HR** | **p** |
| Zero | REFERENCE | | |
| Low | 1.42 | 0.37-5.49 | 0.61 |
| Medium | 2.51 | 0.84-7.49 | 0.10 |
| High | 5.14 | 1.66-15.94 | <0.01 |

*adjusted for: recipient age, recipient gender, recipient race/ethnicity, recipient BMI, dialysis vintage, cause of end stage renal disease, preemptive kidney transplantation, history of prior kidney transplantation, multi-organ transplantation, type of induction therapy, donor age, donor gender, donor race/ethnicity, donor type [living vs. deceased], cold ischemia time, cytomegalovirus risk status, cPRA at allocation

Abbreviations:

CI: confidence Interval

HR: Hazard Ratio

MM: mismatch

**Supplemental Table 6:** Association of risk groups (risk groups based on PIRCHE-T2) and developing de novo DSA using Cox proportional regression models

| **Unadjusted model** | | | |
| --- | --- | --- | --- |
| **Risk Groups** | **De novo DSA** | | |
|  | **HR** | **95%CI of HR** | **p** |
| Low | REFERENCE | | |
| Medium-low | 1.05 | 0.45-2.46 | 0.91 |
| Medium-high | 1.98 | 0.97-4.08 | 0.06 |
| High | 3.65 | 1.71-7.79 | <0.01 |
| **Adjusted model*** | | | |
| **Risk Groups** | **De novo DSA** | | |
|  | **HR** | **95%CI of HR** | **p** |
| Low | REFERENCE | | |
| Medium-low | 1.36 | 0.55-3.35 | 0.50 |
| Medium-high | 2.47 | 1.14-5.36 | 0.02 |
| High | 4.23 | 1.88-9.51 | <0.01 |

*adjusted for: recipient age, recipient gender, recipient race/ethnicity, recipient BMI, dialysis vintage, cause of end stage renal disease, preemptive kidney transplantation, history of prior kidney transplantation, multi-organ transplantation, type of induction therapy, donor age, donor gender, donor race/ethnicity, donor type [living vs. deceased], cold ischemia time, cytomegalovirus risk status, cPRA at allocation

Abbreviations:

CI: confidence Interval

HR: Hazard Ratio

**Supplemental Table 7:** Baseline characteristics of excluded versus included patients in the whole cohort

|  | **Excluded recipients** | **Included recipients** | **p-value** |
| --- | --- | --- | --- |
| **N** | 196 | 493 |  |
| **Recipients’ characteristics** |  |  |  |
| **Age (years), mean (SD)** | 50.7 (14.6) | 49.7 (15.1) | 0.41 |
| **Gender (N (%))** |  |  | 0.83 |
| male | 121 (61.7%) | 300 (60.9%) |  |
| female | 75 (38.3%) | 193 (39.1%) |  |
| **Race/ethnicity (N (%))** |  |  | 0.32 |
| White | 121 (64.4%) | 344 (72.3%) |  |
| Hispanic | 43 (22.9%) | 85 (17.9%) |  |
| African American | 4 (2.1%) | 11 (2.3%) |  |
| Asian | 8 (4.3%) | 18 (3.8%) |  |
| Other | 12 (6.4%) | 17 (3.6%) |  |
| Unknown | 0 (0.0%) | 1 (0.2%) |  |
| **Body Mass Index (kg/m2), mean (SD)** | 28.67 (5.99) | 28.69 (5.57) | 0.98 |
| **Dialysis vintage (months), median (IQR)** | 41.8 (23.8-66.3) | 29.5 (13.7-49.7) | <0.001 |
| **Cause of kidney failure (N (%))** |  |  | 0.43 |
| Diabetes mellitus | 69 (35.2%) | 147 (29.8%) |  |
| Hypertension/Vascular disease | 28 (14.3%) | 59 (12.0%) |  |
| Cystic kidney disease | 13 (6.6%) | 42 (8.5%) |  |
| Glomerulonephritis | 44 (22.5%) | 116 (23.5%) |  |
| Other/unknown | 42 (21.4%) | 129 (26.2%) |  |
| **Prior kidney transplant (N (%))** |  |  | 0.85 |
| No | 176 (89.8%) | 445 (90.3%) |  |
| Yes | 20 (10.2%) | 48 (9.7%) |  |
| **Multi-organ transplantation (N (%))** |  |  | 0.26 |
| Liver + kidney | 3 (1.5%) | 12 (2.4%) |  |
| Pancreas + kidney | 3 (1.5%) | 15 (3.0%) |  |
| Heart + kidney | 1 (0.5%) | 10 (2.0%) |  |
| None | 189 (96.4%) | 453 (91.9%) |  |
| Liver +Heart+Kidney | 0 (0.0%) | 3 (0.6%) |  |
| **CMV risk categories (N (%))** |  |  | 0.28 |
| Low | 51 (26.0%) | 122 (24.7%) |  |
| Intermediate | 75 (38.3%) | 225 (45.6%) |  |
| High | 64 (32.7%) | 130 (26.4%) |  |
| Unknown | 6 (3.1%) | 16 (3.2%) |  |
| **Was the donor organ pumped? (N (%))** |  |  | <0.001 |
| No | 16 (8.2%) | 171 (37.7%) |  |
| Yes | 180 (91.8%) | 282 (62.3%) |  |
| **En block kidney (N (%))** | 3 (7%) | 11 (15%) | 0.20 |
| **Dual kidney (N (%))** | 9 (21%) | 6 (8%) | 0.045 |
| **Transplantation characteristics** |  |  |  |
| **Cold ischemia time (hours), median (IQR)** | 16.3 (9.7-20.6) | 12.1 (3.6-20.2) | <0.001 |
| **cPRA (%), median (IQR)** | 0 (0-3.5) | 0 (0-0) | 0.44 |
| **Donors’ characteristics** |  |  |  |
| **Age (years), mean (SD)** | 37.5 (16.6) | 37.3 (15.4) | 0.87 |
| **Gender (N (%))** |  |  | 0.057 |
| male | 121 (61.7%) | 265 (53.8%) |  |
| female | 75 (38.3%) | 228 (46.2%) |  |
| **Race/ethnicity** |  |  | 0.010 |
| White | 174 (88.8%) | 385 (78.1%) |  |
| African American | 3 (1.5%) | 18 (3.7%) |  |
| Asian | 5 (2.6%) | 9 (1.8%) |  |
| Other | 2 (1.0%) | 8 (1.6%) |  |
| Unknown | 12 (6.1%) | 73 (14.8%) |  |
| **Donor type (N (%))** |  |  | <0.001 |
| Living | 11 (5.6%) | 202 (41.0%) |  |
| Deceased | 185 (94.4%) | 291 (59.0%) |  |
| **Donor DCD (N (%))** |  |  | 0.071 |
| No | 97 (52.4%) | 177 (60.8%) |  |
| Yes | 88 (47.6%) | 114 (39.2%) |  |
| **KDPI, median (IQR)** | 36 (18-55) | 23 (10-52) | <0.001 |
| **Donor cause of death (N (%))** |  |  | 0.091 |
| Anoxia | 104 (56.2%) | 131 (45.0%) |  |
| Cerebrovascular/stroke | 26 (14.1%) | 41 (14.1%) |  |
| Head trauma | 50 (27.0%) | 106 (36.4%) |  |
| Central nervous system tumor | 0 (0.0%) | 3 (1.0%) |  |
| Other | 5 (2.7%) | 10 (3.4%) |  |
| **Donor terminal creatinine (mg/dl), median (IQR)** | 0.83 (0.63-1.11) | 0.81 (0.65-1.00) | 0.20 |
| **Donors diabetes (N (%))** |  |  | 0.068 |
| No | 180 (92.8%) | 469 (96.1%) |  |
| Yes | 14 (7.2%) | 19 (3.9%) |  |
| **Donors hypertension (N (%))** |  |  | <0.001 |
| No | 144 (74.6%) | 432 (89.1%) |  |
| Yes | 49 (25.4%) | 53 (10.9%) |  |
| **Donor malignancy (N (%))** |  |  | 0.95 |
| No | 191 (97.4%) | 480 (97.4%) |  |
| Yes | 5 (2.6%) | 13 (2.6%) |  |
| **Immunological characteristics** |  |  |  |
| **Number of HLA mismatches (HLA A,B and DR) (N (%))** |  |  | <0.001 |
| 0 | 3 (1.5%) | 23 (4.7%) |  |
| 1 | 2 (1.0%) | 15 (3.0%) |  |
| 2 | 5 (2.6%) | 35 (7.1%) |  |
| 3 | 43 (21.9%) | 79 (16.0%) |  |
| 4 | 52 (26.5%) | 114 (23.1%) |  |
| 5 | 73 (37.2%) | 142 (28.8%) |  |
| 6 | 18 (9.2%) | 85 (17.2%) |  |
| **Induction treatment (N (%))** |  |  |  |
| No Induction | 0 (0%) | 0 (0%) |  |
| Basiliximab | 18 (9.2%) | 66 (13.4%) | 0.13 |
| Thymoglobulin | 173 (88.3%) | 399 (80.9%) | 0.021 |
| Steroid | 196 (100.0%) | 492 (99.8%) | 0.53 |
| Alemtuzumab | 3 (1.5%) | 18 (3.7%) | 0.14 |
| **Outcomes** |  |  |  |
| **Proportion of Antibody Mediated Rejection of the subset of patient underwent biopsy (N (%))** |  |  | 0.052 |
| No | 64 (83.1%) | 121 (71.6%) |  |
| Yes | 13 (16.9%) | 48 (28.4%) |  |
| **Proportion of rejection of the subset of patient underwent biopsy (N (%))** |  |  | 0.78 |
| No | 47 (61.0%) | 100 (59.2%) |  |
| Yes | 30 (39.0%) | 69 (40.8%) |  |
| **Proportion of T-cell Mediated Rejection of the subset of patient underwent biopsy (N (%))** |  |  | 0.85 |
| No | 52 (67.5%) | 112 (66.3%) |  |
| Yes | 25 (32.5%) | 57 (33.7%) |  |
| **Post-Transplant Donor Specific Antibodies (N (%))** |  |  | 0.14 |
| No | 136 (72.7%) | 379 (78.1%) |  |
| Yes | 51 (27.3%) | 106 (21.9%) |  |
| **Dd-cf-DNA elevation (N (%))** |  |  | 0.26 |
| No | 107 (75.9%) | 312 (80.4%) |  |
| Yes | 34 (24.1%) | 76 (19.6%) |  |
| **Primary outcome (N (%))** |  |  | 0.017 |
| No | 107 (56.3%) | 326 (66.1%) |  |
| Yes | 83 (43.7%) | 167 (33.9%) |  |
| **Delayed Graft Function (N (%))** |  |  | 0.56 |
| No | 177 (90.3%) | 452 (91.7%) |  |
| Yes | 19 (9.7%) | 41 (8.3%) |  |
| **Death (N (%))** |  |  | 0.004 |
| No | 179 (91.3%) | 476 (96.6%) |  |
| Yes | 17 (8.7%) | 17 (3.4%) |  |
| **Graft Loss (N (%))** |  |  | 0.88 |
| No | 190 (96.9%) | 479 (97.2%) |  |
| Yes | 6 (3.1%) | 14 (2.8%) |  |

Values are expressed as mean (standard deviation), median (interquartile range), or number (%). Continuous variables were compared via t-tests or Mann-Whitney U tests. Categorical variables were compared via Chi-square tests.

**Legend of Supplementary Figures**

**Figure S1:** Kaplan-Meier curves by four Eplet groups for outcomes (Panel A: Early Graft Injury outcome; Panel B: DSA outcome; Panel C: Rejection outcome; Panel D: Elevation of dd-cfDNA outcome)

**Figure S2:** Kaplan-Meier curves by risk groups (risk groups based on PIRCHE-T2 score) for outcomes (Panel A: Early Graft Injury outcome; Panel B: DSA outcome; Panel C: Rejection outcome; Panel D: Elevation of dd-cfDNA outcome)

**Figure S3:** Kaplan-Meier curves for de novo DSA-free outcomes (Panel A: four eplet categories; Panel B: three eplet categories; Panel C: risk groups based on PIRCHE-T2 and PIRCHE-B scores; Panel D: risk groups based on PIRCHE-T2 score)

**Figure S1:**

**Figure S2:**

**Figure S3:**
